# A Comparison of Binary and Integer Encodings in Genetic Algorithms for the Maximum *k*-Coverage Problem with Various Genetic Operators

**DOI:** 10.3390/biomimetics10050274

**Published:** 2025-04-28

**Authors:** Yoon Choi, Jingeun Kim, Yourim Yoon

**Affiliations:** 1 Department of Computer Science, Yonsei University, 50 Yonsei-ro, Seodaemoon-gu, Seoul 03722, Republic of Korea; unidentity95@yonsei.ac.kr; 2 Department of IT Convergence Engineering, Gachon University, 1342 Seongnam-daero, Sujeong-gu, Seongnam-si 13120, Gyeonggi-do, Republic of Korea; wlsrms27@gachon.ac.kr; 3 Department of Computer Engineering, Gachon University, 1342 Seongnam-daero, Sujeong-gu, Seongnam-si 13120, Gyeonggi-do, Republic of Korea

**Keywords:** maximum k-coverage problem, genetic algorithm, combinatorial optimization, binary encoding, integer encoding

## Abstract

The maximum *k*-coverage problem (MKCP) is a problem of finding a solution that includes the maximum number of covered rows by selecting *k* columns from an *m* × *n* matrix of 0s and 1s. This is an NP-hard problem that is difficult to solve in a realistic time; therefore, it cannot be solved with a general deterministic algorithm. In this study, genetic algorithms (GAs), an evolutionary arithmetic technique, were used to solve the MKCP. Genetic algorithms (GAs) are meta-heuristic algorithms that create an initial solution group, select two parent solutions from the solution group, apply crossover and repair operations, and replace the generated offspring with the previous parent solution to move to the next generation. Here, to solve the MKCP with binary and integer encoding, genetic algorithms were designed with various crossover and repair operators, and the results of the proposed algorithms were demonstrated using benchmark data from the OR-library. The performances of the GAs with various crossover and repair operators were also compared for each encoding type through experiments. In binary encoding, the combination of uniform crossover and random repair improved the average objective value by up to 3.24% compared to one-point crossover and random repair across the tested instances. The conservative repair method was not suitable for binary encoding compared to the random repair method. In contrast, in integer encoding, the combination of uniform crossover and conservative repair achieved up to 4.47% better average performance than one-point crossover and conservative repair. The conservative repair method was less suitable with one-point crossover operators than the random repair method, but, with uniform crossover, was better.

## 1. Introduction

The maximum k-coverage problem (MKCP) is a type of set-covering problem. When matrix A = $\left(a_{ij}\right)$ is a binary matrix with a size of m×n, the purpose of the MKCP is to select *k* columns covering the m×n matrix such that the number of rows contained is at its maximum [1,2]. The MKCP has applications in many engineering fields, such as the maximum covering location problem [3], cloud computing [4], blog-watch [5], influence maximization problems for target marketing [6], and optimizing recommendation system in e-commerce [7].

The MKCP is famous for its NP-hardness [8]. GAs do not guarantee finding an optimal solution for NP-hard problems in polynomial time. However, they are well known for providing a reasonably better solution in a suitable time than heuristic and deterministic algorithms for optimization [9].

The solution for the MKCP in GAs can be expressed in a one-dimensional array whose elements present whether the corresponding column is selected or not. In integer encoding, the element of the array itself indicates whether that column is selected or not. In binary encoding, the element has only a value of 1 or 0. The value of 1 means the corresponding column is selected, and the value of 0 means it is not selected.

**Figure 1:**
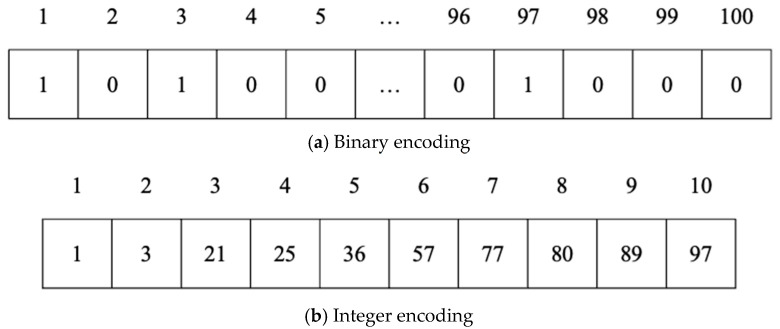
Examples of binary and integer encodings for the MKCP.

Figure 1 shows an example of a binary encoding and integer encoding, where A is a 2000×100 matrix and the value of *k* is 10. In binary encoding, an array for a solution contains only 0 and 1. The size of the array is 100 because the number of columns of the matrix is 100. The value of 1 means that the column corresponding to the index of the element is selected for the solution. In integer encoding, the size of an array for a solution is 10 because the value of *k* is 10. The value of each element in the array means that the corresponding column is selected. Because of these characteristics, there is a difference between binary encoding and integer encoding.

The performances of GAs for the MKCP with the two encodings and genetic operators were examined in this study. Through the experiments, the best combinations of encodings and operators could be derived. Genetic algorithms were designed with two different encoding methods, binary and integer encodings. In the proposed GAs, two solutions composed of *k* columns are randomly selected from a solution group, which is called a population. An offspring solution is generated by sequentially applying crossover, repair, and replacement operations. After a predetermined number of generations, the solution with the maximum target value is selected as the final solution. The performances of the proposed methods, which are based on binary and integer encodings, with various crossover operators and repair feasibilities, are compared and verified [10,11].

In this study, GAs for solving the MKCP are presented in detail, and various encodings and genetic operators are investigated. The effectiveness of GAs for solving the MKCP could be shown through the experiments. This study also showed which combination of encodings and genetic operators is more effective in solving the MKCP.

The organization of the paper is as follows: Section 2 reviews the related works in the field. Section 3 defines the problem being addressed. Section 4 details the genetic algorithm (GA) used for the MKCP in this study. Section 5 presents and analyzes the experimental results. Finally, Section 6 concludes the paper and offers suggestions for future directions.

## 2. Related Works

The MKCP is a combinatorial problem and there have been several theoretical studies on the generalizations and algorithms for solving the problem [12]. The purpose of the maximum coverage problem is to select *k* sets so that the weight of the covered element is maximized. There is a previous study that solves the MKCP with a standard greedy algorithm by Hochbaum and Pathria [13]. The algorithm proposed in the paper adds sets repeatedly so that the weight is maximized when adding sets. In [13], it was proved that the proposed greedy algorithm has an approximation ratio of $\left(1-\frac{1}{e}\right)$. Nemhauser et al. [14] tried to solve a more generalized version of the MKCP. They proposed an approximation algorithm for the problem and obtained the same approximation ratio. Although it is already well known that the MKCP is a type of NP-hard problem, Feige [15] proved the inapproximability of the problem. In other words, when P = NP is not true, the algorithm for solving the MKCP cannot have a better approximation ratio than ($1-\frac{1}{e}$). Resende suggested another way to solve the MKCP. A greedy randomized adaptive search procedure (GRASP) was used as a heuristic algorithm [16]. The upper bound for optimal values was obtained by considering a linear programming relaxation. GRASP performed better than the greedy algorithm, but no theoretical proof of GRASP was made. Later, the constrained MKCP was studied by Khuller et al. [17]. Instead of limiting the size of the solution, each set had a cost and was made available only if it was less than a predetermined cost. To calculate the approximation ratio, Khuller et al. [17] devised two algorithms. The first one achieved an approximation ratio of $\frac{1}{2}(1-\frac{1}{e})$ and the second one achieved an approximation ratio of $\left(1-\frac{1}{e}\right)$. In addition, they demonstrated that under similar conditions proposed by Feige [15], the highest possible approximation ratio for the constrained MKCP is $\left(1-\frac{1}{e}\right)$.

The MKCP is an NP-hard problem [8]. It is strongly suspected that there are no polynomial-time algorithms for NP-hard problems, even though that has not been proven thus far. Hence, it is nearly impossible to find the optimal solution in practical time. To solve this kind of problem, heuristic algorithms are usually applied. Heuristic algorithms may not find the optimal solution, but they try to find reasonably good solutions in practical time.

The genetic algorithm is also a meta-heuristic algorithm. It can be applied to many NP-hard problems as well as the MKCP. The encoding scheme is a very important factor in GAs. The given information must be encoded into a specific bit string and there have been various encoding schemes according to characteristics of problems [18,19]. Binary encoding is one of the most common encoding schemes. Payne and Glen [20] developed a GA based on binary encoding to identify similarities between molecules. In their research, binary encoding is used for the position and shape of molecules. Longyan et al. [21] studied three different methods of using a binary-encoded GA for wind farm design. Although binary encoding is the most common encoding scheme, in some cases, the gene or chromosome is represented using a string of some values [22]. These values can be real, integer numbers, or characters.

Among various genetic operators, the crossover is considered the most critical genetic operator in GAs. The crossover operator is used to create offspring by mixing information from two or more parents. Some well-known crossover operators are one-point, two-point, k-point, and uniform crossovers [23]. In one-point crossover, one point is randomly selected. From that point, the genetic information of the two parents is exchanged. In two-point and k-point crossover, two or more random points are selected, and the parents’ genetic information is exchanged from the selected point as in the one-point crossover. In uniform crossover, specific points are not selected for the exchange of genetic information. Instead, for each gene it is randomly determined whether it will be exchanged or not.

MKCP is deeply connected to the minimum set covering problem (MSCP), which is a frequently researched NP-hard combinatorial optimization problem. Meta-heuristics, such as tabu search [24], ant colony optimization [25], and particle swarm optimization [26], have been applied to the MSCP, but the MKCP has been less studied [8]. The techniques used in the MSCP are challenging to apply directly to the MKCP because the two problems have slightly different structures. For solving the MKCP, there is previous research using adaptive binary particle swarm optimization, which is a representative meta-heuristic algorithm [27]. In other research, the method of combining the ant system with effective local search has been proposed [28]. Both studies focused on effective applications of meta-heuristic algorithms to solve the MKCP. In this paper, GAs were used to solve the MKCP. The performances of GAs with various genetic operators are investigated and analyzed. This study can be used as a guide for designing GAs to solve the MKCP efficiently.

Recent studies have further expanded the design of genetic operators to enhance the scalability and robustness of GAs, especially in large-scale optimization settings. For instance, Akopov [29] proposed a matrix-based hybrid genetic algorithm (MBHGA) for solving agent-based models, integrating real and integer encodings with hybrid crossover mechanisms to improve convergence speed and accuracy in multi-agent systems. Deb and Beyer [30] introduced a self-adaptive GA using simulated binary crossover (SBX), which dynamically adjusts the crossover distribution index to balance exploration and exploitation in real-coded optimization problems.

These works demonstrate the flexibility and adaptability of genetic algorithms when combined with carefully designed operators and encoding strategies. Although our current study focuses on classical crossover and repair operators under binary and integer encodings, incorporating more advanced operator schemes like SBX or hybrid frameworks is a promising direction for future research, especially for scaling the MKCP to even larger problem instances.

## 3. Problem Statement

Let $A=\left(a_{ij}\right)$ be an m×n 0–1 matrix and let $w_{i\;}$ be a weight function for each row of matrix A. The challenge of this problem is to select *k* columns for the maximum number of covered rows of matrix A. This can be formulated as follows:

(1) $$maxmize\sum_{i=1}^{m}w_{i}\cdot I\left(\sum_{j=1}^{n}a_{ij}x_{j}\geq 1\right)\;subject\;to\sum_{j=1}^{n}x_{j}=k\;x_{j}\in \left\{0,1\right\},\;\;\;j=1,2,\ldots ,n$$

*I*(·) is an indicator function determining whether 0 (false) or 1 (true) [8]. In this study, we assume that the weights are equal to 1. The fitness of a particular solution was measured according to how many rows of a problem matrix were covered.

In this study, 65 instances of 11 set cover problems from the OR-library [31] were used in our experiments. The details for each dataset are shown in Table 1. In Table 1, each number of rows and columns is represented by *m* and *n*, respectively. In previous research on the MKCP [10], values of *k* were fixed as 10 and 20. However, here, the values of *k* using the tightness ratio were determined in the same way as in [8]; the high tightness ratio means the largest optimal value [9]. Each *k* value corresponding to a tightness ratio is described in Table 2. To apply genetic algorithms to each data instance, 400 individuals were generated by randomly selecting *k* columns for an initial population of genetic algorithms.

## 4. Encoding and Genetic Operators for the MKCP

Genetic Algorithms (GAs) are a class of meta-heuristic algorithms inspired by the process of natural selection. They are widely used for solving combinatorial and NP-hard problems due to their ability to efficiently explore large search spaces.

A typical GA starts by generating an initial population of candidate solutions. In each generation, individuals from the current population are selected based on their fitness, and new solutions (offspring) are generated by applying crossover (recombination) and mutation operations. The offspring then replace some or all individuals in the current population, depending on the replacement strategy. This process continues for a fixed number of generations or until a convergence criterion is met.

The performance of a GA heavily depends on the encoding of solutions, the choice of genetic operators (crossover, mutation, repair), and parameter settings such as population size and number of generations. In this study, we focus on encoding and operator choices tailored to the maximum *k*-coverage problem (MKCP), and the mutation operation is partially integrated into the repair process.

### 4.1. GA Encodings for the MKCP

The choice of binary and integer encodings is closely aligned with the structure of the MKCP. In binary encoding, the representation naturally supports fixed-length chromosomes with element-wise selection flags, making it compatible with standard crossover and mutation techniques in genetic algorithms. Integer encoding, on the other hand, directly represents a solution by listing the *k* selected column indices, which reduces redundancy and allows for more compact representation, especially in problems where *k* << *n*.

These two encoding methods have been commonly employed in GA-based studies of combinatorial optimization problems, including feature selection, facility location, and routing [18,20,22]. Their simplicity and effectiveness make them suitable candidates for benchmarking GA performance on the MKCP. In this study, we systematically evaluate both encodings under controlled experimental settings.

In integer encoding, a solution is represented with integers, which correspond to the indices of selected rows, while in binary encoding, a solution is represented by 1s and 0s, with 1 indicating that the corresponding row is selected, and 0 implying it is not [8].

In integer encoding, the length of a solution array is determined according to the *k* value of the MKCP. For example, if the *k* value of the MKCP is 40, the length of the array of the solution will also be 40. The range of the integers that comprise the array of a solution is determined by the number of columns of the matrix to be solved. If the number of columns of the matrix is 2000, a correct solution is generated with integers from 1 to 2000.

In binary encoding, the length of an array of a solution is determined according to the number of columns of the matrix to be solved. The value of *k* determines the number of 1s in a solution; for example, a *k* value of 40 implies 40 1s in a solution. A value of 1 in a solution implies selecting the column corresponding to the index.

### 4.2. Crossover Operators for the MKCP

The genetic algorithm is an evolutionary arithmetic technique and is an algorithm technique that finds an optimal solution by imitating the evolutionary process of nature. A typical GA determines the optimum value of a problem function based on a repetitive process of recombination of two parent solutions to create an offspring solution [8].

**Figure 2:**
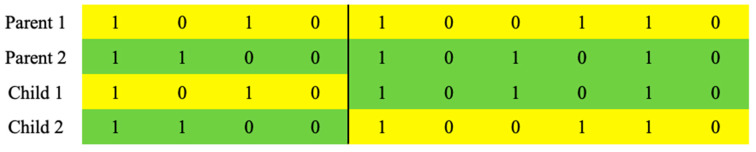
An example of one-point crossover.

In this study, the number of the population of GAs was set to 400; to create child solutions, four-hundred solutions in the population were randomly paired, and one-point and uniform crossovers, which are representative traditional crossovers of genetic algorithms, were used as crossover operators. The performances of binary and integer encodings were analyzed with these crossover operators. A one-point crossover operator generates a child solution by copying another part of two parent solutions, based on point p, which is selected randomly from the one-dimensional array of a chromosome with length n. An example of this process is presented in Figure 2.

In Figure 2, yellow cells represent genes inherited from Parent 1, and green cells represent genes inherited from Parent 2. A value of 1 indicates that a corresponding column is selected, and a value of 0 indicates that it is not selected. In the figure, point p between the 4th and 5th genes is selected as a crossover point. For our study, only Child 1 was adopted, and Child 2 was not considered. The entire number of possible crossover points in one-point crossover is n−1 [32].

**Figure 3:**
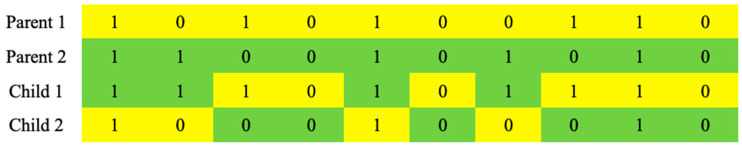
An example of uniform crossover.

A uniform crossover operator generates an offspring solution by selecting each gene from either parent with equal probability [2]. That is, for each index, there is a 50 percent probability of which parent to select. In the uniform crossover, chromosomes are not divided into segments as they are in one-point crossover [33]. Each gene is dealt with separately. Again, in our GAs, only Child 1 was adopted, and Child 2 was not considered. An example of the process is presented in Figure 3. In the figure, yellow cells represent genes inherited from Parent 1, and green cells represent genes inherited from Parent 2. A value of 1 indicates that a corresponding column is selected, and a value of 0 indicates that it is not selected.

In [34], Bolotbekova et al. employed a crossover ensemble method to explore the most suitable combination. Similarly, we conducted experiments with various crossover combinations to observe performance tendencies, with the results presented in Appendix A.

### 4.3. Repair Operators for the MKCP

**Figure 4:**
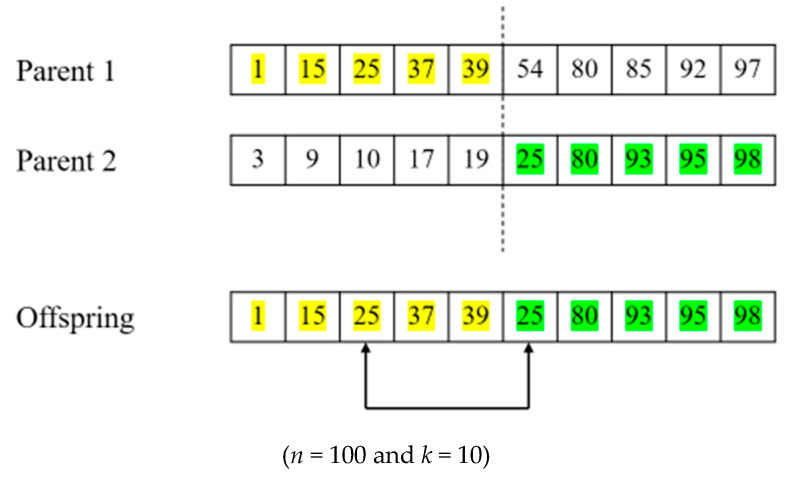
How infeasible solutions can be generated after one-point crossover. Yellow cells represent genes inherited from Parent 1, and green cells represent genes inherited from Parent 2.

After both one-point and uniform crossover operations, the generated offspring may be infeasible, that is, it does not satisfy the constraint on the MKCP [35]. In this case (Figure 4), a repairing phase can operate to reinstate feasibility, and this makes it possible to perform a part of mutation functions.

**Figure 5:**
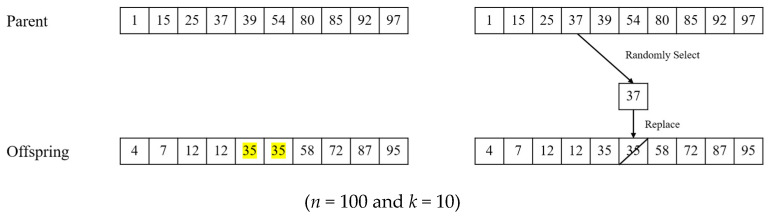
An example of a conservative repair operation.

Figure 5 presents an example of the process of a conservative repair operation in integer encoding. In Figure 5, the offspring has a duplicate value of 35, which makes the solution infeasible. A conservative repair operator takes a gene from parent solutions and replaces one of the duplicated genes of the offspring with the gene from a parent. In this process, the gene is randomly selected from a parent solution. Figure 5 shows that a value of 37 is selected from a parent solution, and one of the duplicated genes with a value of 35 is replaced with 37. The repair operator repeats this process until the constraint on the MKCP is satisfied.

A random repair operator performs the entire process similarly to a conservative repair operator [36], the difference being that replacements for duplicated values are not taken from parent solutions, but randomly selected among all the possible values. Both repair operators can have the function of mutation. Therefore, in this study, the mutation process of the genetic algorithm was partially replaced by a repair operation, and a separate mutation operator was not applied.

In this study, a separate mutation operator was not applied. Instead, mutation-like behavior was embedded within the repair process. Specifically, the random repair operator introduces diversity by randomly selecting replacement values when a solution becomes infeasible after crossover. This mechanism mimics the role of mutation in maintaining genetic variation without explicitly invoking a dedicated mutation step.

Although the mutation probability was not separately defined, this effect is triggered every time an infeasible solution is repaired—effectively operating at a frequency determined by the rate of infeasible offspring generation. This design choice simplifies the algorithm while ensuring that exploration is preserved. Future work may further investigate the explicit integration of advanced mutation operators alongside repair-based correction.

### 4.4. Genetic Framework for the MKCP

**Figure 6:**
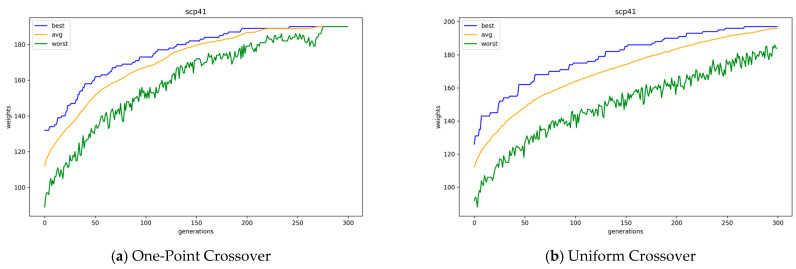
Convergence of GAs with one-point and uniform crossover operators in binary encoding.

Before each offspring generation, a population of 400 parent solutions is randomly paired to generate 200 offspring solutions. This population size of 400 is commonly used when solving the MKCP problem using GAs to cover the large search space of this problem [2]. The generated offspring solutions are exchanged with 200 parent solutions with the least objective function values. The best solution and its objective value obtained over 300 generations are recorded as the result of the performed GA. Figure 6 is the pseudocode of the GA used in our experiment.

Initially, a population of solutions is randomly generated, and after a predetermined number of generations (300), the GA is terminated. In a generation, solutions are randomly paired, and for each pair, an offspring is produced by crossover and repair operators with 100% probability [2]. Repair operators are applied only when the produced offspring is not feasible, that is, it does not satisfy the constraint on the MKCP. Next, the newly generated offspring are evaluated, and solutions in the previous population are sorted according to their objective values. Half the previous population with low fitness is replaced by new offspring. Based on the genetic framework in Algorithm 1, different crossover and repair operators are applied and compared in our experiments [8].
**Algorithm 1**: Pseudocode of Genetic Algorithm Used in this Study**Input**: Population size *N*, Number of generation maximum_generation **Output**: Best individual Initialize a population *P* of *N* individuals **// Ensure initial population satisfies the *k*-column constraint (exactly *k* columns selected)**
**for** i = 1 to maximum_generation do Randomly form *N*/2 pairs from population *P* offspring_list ← ∅ **for** each pair (p1, p2) do  offspring = crossover(p1, p2)  **if** (constraint is not satisfied) then   **while** (constraint is not satisfied) do    repair(offspring)   **end** while  **end** if ** // No separate mutation step; repair includes random replacement to induce diversity** offspring_list ← offspring_list ∪ offspring **end** for evaluate fitness values of offspring_list sort *P* by fitness values in descending order Replace the bottom half of P with offspring_list **end** for Return the best individual in *P* Return the best individual in *P*

## 5. Experimental Results

### 5.1. Experimental Settings

Table 2 shows the size of the data used in the experiments; the experiments were conducted by setting the *k* value differently for each of the number of rows and columns of the corresponding matrix for the MKCP.

A total of eight experiments were conducted with two types of encoding methods (binary encoding, integer encoding), two types of crossover operators (one-point crossover, uniform crossover), and two types of repair operators (conservative repair, random repair). In all experiments, the number of populations was fixed at 400 and the number of generations was fixed at 300. In each generation, half the population with low fitness value was replaced by child solutions that had been generated based on 200 pairs of parent solutions. Parent solutions were randomly paired before crossover operations. For each experiment, 30 runs of GAs were performed, and the average objective value over 30 runs with average running time was recorded. Each experiment was conducted on all tightness ratios. The experiments were run on a computer equipped with a 3.2 GHz AMD Ryzen 7 5800H processor (Advanced Micro Devices, Santa Clara, CA, USA) and 16 GB of RAM. The proposed genetic algorithms were implemented using the Java programming language.

### 5.2. Results with Binary Encoding

Table 3 presents the average for each best objective value and running time of 30 runs of GAs with binary encoding. As shown in Table 3, uniform crossover operations show better results than those of one-point crossover. This characteristic manifests regardless of the type of repair operation. In particular, the larger the instance size and *k* value, the larger the performance difference between the two crossover methods.

In Figure 6, the average, best, and worst objective values of the GA solution according to the generation are plotted with two types of crossover operators for problem instance Scp41, which is an instance of the problem set Scp4x. In Figure 6, random repair was applied, and it shows that one-point crossover operations converge slightly faster than uniform crossover operations. In the case of one-point crossover operations, the average and best values already became similar from approximately the 150th generation, whereas, in the case of uniform crossover operations, they did not converge until approximately the 300th generation. In addition, by examining the worst-performing individuals within the population, it can be observed that the overall quality of individuals is higher when using a one-point crossover compared to a uniform crossover.

Differences in performance results according to repair methods can also be seen in Table 3. Comparing conservative repair and random repair, conservative repair showed worse performance than random repair in binary encoding. The running times of GAs with the conservative repair method were slightly faster than that of GAs with the random repair method; however, the solution quality of the conservative repair method, according to objective values, was significantly lower than that of the random repair method. The average performance of the combination of uniform crossover and random repair improved by up to 3.24% compared to the combination of one-point crossover and random repair. Furthermore, we conducted Welch’s t-test to compare the performance of different crossover methods under the same repair strategy. The results statistically validate that the use of uniform crossover yields significantly better performance than one-point crossover, regardless of the repair method applied.

The random repair method has a slightly larger mutation effect than the conservative repair method. Hence, the convergence of the solution population is slow when using the random repair method. That is, the random repair method can maintain more diversity in solutions than the conservative repair method. Similar to the comparative results of crossover operations, maintaining diversity without rapid convergence is advantageous for solving the MKCP.

### 5.3. Results with Integer Encoding

**Figure 7:**
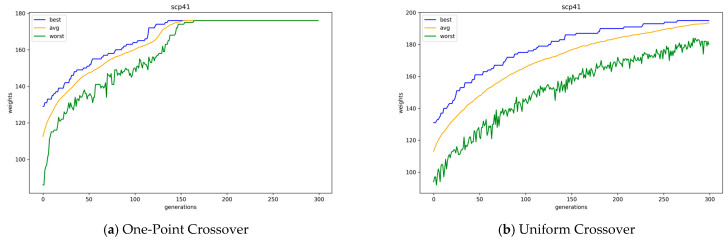
Convergence of GAs with one-point and uniform crossover operations in integer encoding.

Table 4 presents the average for each best objective value and running time of 30 runs of GAs with integer encoding. In the table, the combination of uniform crossover operations and the conservative repair method has the best results overall. However, in cases with the random repair method, one-point crossover operations show better results when the *k* value is large, while uniform crossover operations show better results when the *k* value is small. Figure 7 presents the average, best, and worst objective values of the GAs solution population according to the offspring generation with one-point and uniform crossover operations for problem instance scp41, where the random repair method is used. Here, as in binary encoding, GAs with one-point crossover operations converge slightly faster than GAs with uniform crossover operations. According to an example case in Figure 7, GAs with one-point crossover operations converge completely at the 150th generation, whereas GAs with uniform crossover operations do not converge even at the 300th generation. Moreover, in the case of uniform crossover operations, the best value continuously increases until just before the end of the runs of GAs. Moreover, the worst objective value of the GA with one-point crossover eventually reaches the average objective value at the 164th generation, while the GA with uniform crossover fails to do so. In Table 4, the results of one-point crossover operators are better when the *k* value is large simply because the GAs with uniform crossover operations have not yet converged to the optimal value. The average performance of the combination of uniform crossover and conservative repair improved by up to 4.47% compared to that of one-point crossover and conservative repair. Also, we conducted Welch’s *t*-test to evaluate the performance of different crossover methods under the same repair strategy. The results statistically confirm that uniform crossover yields better performance than one-point crossover, regardless of the repair method applied, except for the case of scpnrfx with tightness ratios of 0.4 and 0.6.

According to Table 4, the conservative repair method was worse than the random repair method when one-point crossover operators were used. However, the conservative repair method showed better results than the random repair method when uniform crossover operators were used. The higher the *k* value, the larger the result value difference between the two repair methods. For example, when the *k* value is 4 or 8 (instance set scpnrex or scpnrfx with tightness ratio 0.8), there is a very small difference; however, when the *k* value is 40 (instance sets scp4x, scp5x, scpcx, and scpnrgx with tightness ratio 0.8), there is a large difference. In the integer encoding, the combination of the conservative repair method and uniform crossover operations showed the best performance.

**Figure 8:**
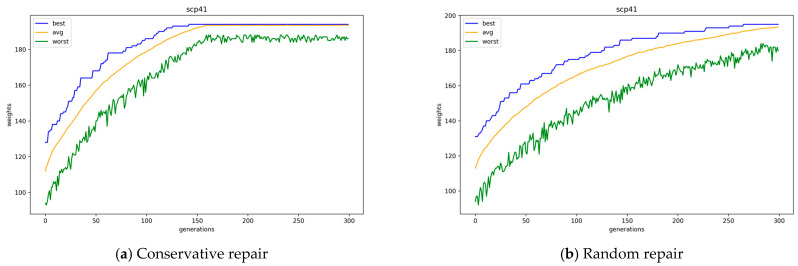
Convergence of GAs with conservative and random repair methods.

Figure 8 presents the average, best, and worst objective values of GA population, according to the offspring generation, with conservative and random repair methods for problem instance scp41, where uniform crossover operators are used. Here, the GAs with the conservative repair method converge a little faster than GAs with the random repair method. The conservative repair method not only converges faster, but also finds a better solution than the random repair method when uniform crossover operators are used. The conservative repair demonstrates more stable improvement in the worst objective values and achieves earlier convergence compared to the random repair.

### 5.4. Comparison of the Performance of Binary and Integer Encodings for the MKCP

**Figure 9:**
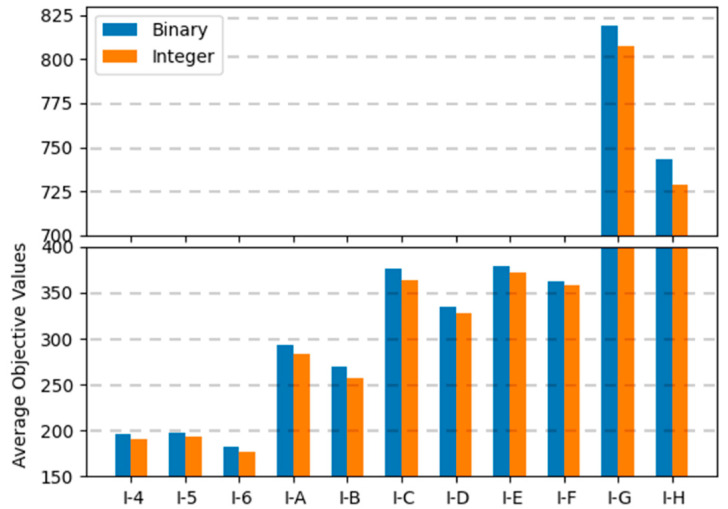
Average objective values of two encodings for instance sets with a tightness ratio of 0.8.

**Figure 10:**
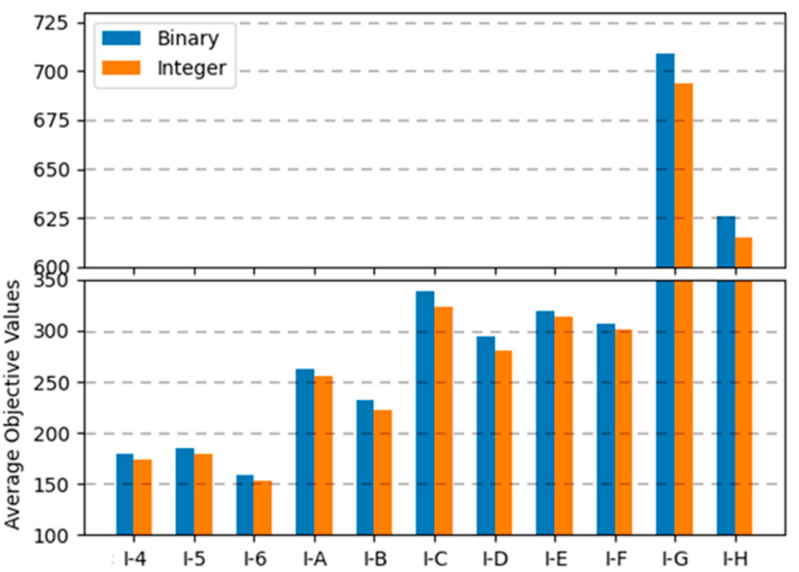
Average objective values of two encodings for instance sets with a tightness ratio of 0.6.

**Figure 11:**
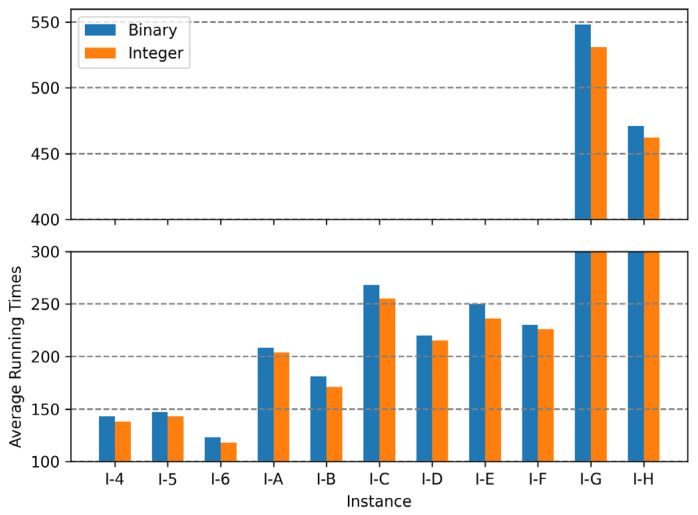
Average objective values of two encodings for instance sets with a tightness ratio of 0.4.

In binary encoding, the combination of uniform crossover operations and the random repair method was the best among the range of combinations; the conservative repair method did not show significant performance variations, and one-point crossover operations were not appropriate for finding optimal solutions for the MKCP. In integer encoding, the combination of uniform crossover operations and the conservative repair method was the best. The two methods that showed the best results for each encoding are compared in graphs in Figure 9, Figure 10 and Figure 11. Comparing all three graphs, they show a consistent trend regardless of the tightness ratio. Although there are differences in objective values according to the tightness ratio, binary encoding showed better performance.

**Figure 12:**
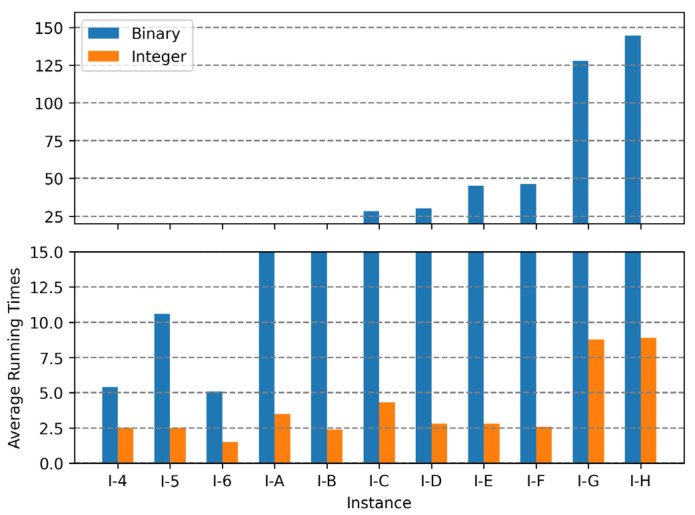
Average running times of two encodings for instance sets with a tightness ratio of 0.8.

**Figure 13:**
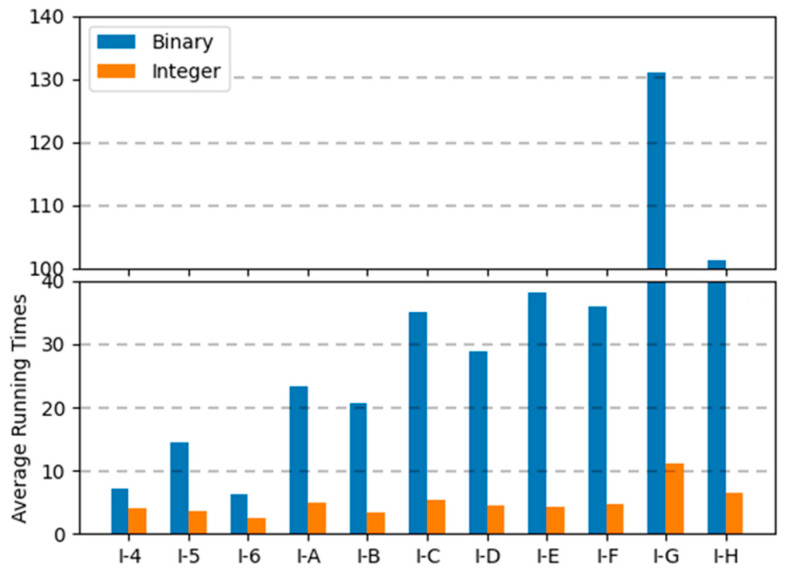
Average running times of two encodings for instance sets with a tightness ratio of 0.6.

**Figure 14:**
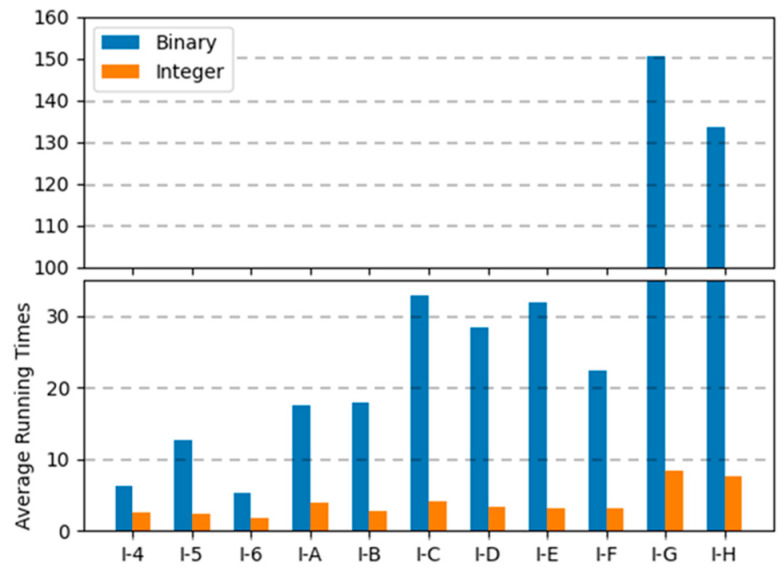
Average running times of two encodings for instance sets with a tightness ratio of 0.4.

Figure 12, Figure 13 and Figure 14 are the graphs of average running times results for each encoding. These figures show more dramatic differences than the graphs of objective values. With integer encoding, the GAs terminated in less than 10 s regardless of the tightness ratio and the size of the instance. In contrast, with binary encoding the run time for the GAs increased with the size of the problem instance set. The tightness ratio did not have a significant impact on the run times for the GAs with binary encoding. In binary encoding, the length of a solution did not change according to the tightness ratio. However, with integer encoding, the smaller the tightness ratio is, the shorter the length of a solution; therefore, GAs for problem sets with a tightness ratio of 0.4 demonstrated shorter run times than those for problem sets with a tightness ratio of 0.8.

## 6. Discussion

In our experiments, we evaluated the performance of genetic algorithms using two types of encoding (binary encoding and integer encoding), two crossover operators (one-point crossover and uniform crossover), and two repair strategies (conservative repair and random repair) across various problem sizes. For binary encoding, uniform crossover consistently outperformed one-point crossover, regardless of the repair strategy. Notably, the combination of uniform crossover and random repair always yielded better objective values than conservative repair. In one instance with a tightness ratio of 0.8, this combination achieved the best objective value of 819.97 for the f instance. This improvement was statistically significant compared to one-point crossover, with a *p*-value of 3.29 × 10^−20^. Similar trends were observed for instances with a tightness ratio of 0.6, where uniform crossover combined with random repair consistently delivered superior performance. For instances with a tightness ratio of 0.4, the performance of uniform crossover with either conservative or random repair was comparable, and both combinations outperformed one-point crossover. Although uniform crossover generally resulted in statistically significant improvements over one-point crossover, an exception was observed for the Scpnrfx instance, where the *p*-value was 2.63 × 10^−1^, indicating a lack of statistical significance (*p* < 0.05).

For integer encoding, the use of conservative repair in conjunction with uniform crossover consistently led to the best performance. In fact, this combination achieved the best results in 30 out of 33 instances, outperforming all other crossover and repair combinations. These findings suggest that, while uniform crossover is generally advantageous, the choice of repair strategy may depend on the encoding type and the specific characteristics of the problem instance. In the case of a tightness ratio of 0.8, the combination of uniform crossover and conservative repair consistently outperformed the combination of one-point crossover and conservative repair across all instances. For a tightness ratio of 0.6, results for the Scpnrfx dataset showed that uniform crossover with conservative repair achieved an objective value of 301.95, which was slightly better than the 301.25 obtained with one-point crossover. However, this difference was not statistically significant, as indicated by a *p*-value of 5.35 × 10^−1^. When the tightness ratio was reduced to 0.4, the performance gap between repair strategies became negligible. For example, in the Scpbx instance, uniform crossover with conservative repair resulted in an objective value of 171.34. The use of random repair in this case yielded a very similar value of 171.36. Both approaches outperformed one-point crossover. Similarly, in the Scpnrex instance, random repair again outperformed conservative repair. Random repair achieved a marginally better result of 236.29, compared to 236.19 obtained with conservative repair. Although the difference between the two repair strategies in this case was minimal, it still suggests a slight edge for random repair. These results show that conservative repair not only outperformed random repair, but also achieved the best performance when combined with uniform crossover. However, the magnitude and statistical significance of this advantage varied depending on the tightness ratio and the specific problem instance.

## 7. Conclusions

In binary encoding, uniform crossover operations showed better results than those of one-point crossover. This is because one-point crossover operations cannot maintain a sufficient diversity of solutions, as the offspring from one-point crossover operations are biased toward a parent solution. Additionally, the conservative repair method was not suitable for finding optimal solutions for the MKCP compared to the random repair method.

In integer encoding, the combination of uniform crossover operation and conservative repair method showed the best results overall. However, in cases of the random repair method, the crossover operation with better performance depended on the value of *k*; when *k* was small, uniform crossover operations found better solutions than those of one-point crossover, and when *k* was large, one-point crossover operations found better solutions. These results are because, with uniform crossover operations, the GAs did not converge enough for large *k* instances. Therefore, by conducting experiments with more offspring generations, different results can be produced. However, except for the instances with *k* value 40, uniform crossover operations were better than one-point crossover operations overall. The conservative repair method was worse than the random repair method when one-point crossover operators were used. However, with uniform crossover operations, the conservative repair method was better than the random repair method.

Throughout the experiments, one-point crossover operations converged quicker than those of uniform crossover. Uniform crossover operations maintained a diversity of solutions in the population better than that of one-point crossover. Despite the faster convergence of one-point crossover, its lack of diversity often resulted in premature convergence to suboptimal solutions. On the other hand, uniform crossover’s ability to preserve diversity in the population allowed for a more thorough exploration of the search space. Since the search space in binary encoding is discrete, conservative repair, which inherits genes from parent solutions, failed to maintain the diversity of offspring. In contrast, random repair allowed for greater exploration of the search space, resulting in better performance. In integer encoding, random repair satisfies the cardinality constraint *k* by adding new subsets randomly, which can introduce low-quality solutions into the offspring. In contrast, conservative repair tends to preserve subsets inherited from the parent solution, thereby maintaining well-performing components that contribute to better overall solution quality.

In binary encoding, the combination of uniform crossover operations and the random repair method was the best, while in integer encoding, the combination of uniform crossover operations and the conservative repair method was the best. Between the two encoding methods, GA runs with the binary encoding framework showed better performance than GA runs with the integer encoding framework in terms of objective values. Conversely, in terms of time utility, integer encoding was much better because the length of the encoded solution using integer encoding is much shorter than that using binary encoding.

However, this study has several limitations. Although we conducted experiments on various instances, there is a need to investigate a broader range of crossover strategies. In addition, further comparative studies are required to evaluate the effectiveness of the operator combinations used in this work on other types of optimization problems beyond the MKCP, and to assess their applicability to real-world problems.

In this study, the effective combination of encodings and genetic operators for the MKCP was shown; however, the relationship between encodings and genetic operators was not analyzed in detail.

## Figures and Tables

**Table 1:** Dataset used for experiments.

Instance Set	Column	Row
Scp4x	200	1000
Scp5x	200	2000
Scp6x	200	1000
Scpax	300	3000
Scpbx	300	3000
Scpcx	400	4000
Scpdx	400	4000
Scpnrex	500	5000
Scpnrfx	500	5000
Scpnrgx	1000	10,000
Scpnrhx	1000	10,000

**Table 2:** *k* Values according to tightness ratios.

Tightness Ratio	Instance Set	*k*
0.8	Scp4x	40
	Scp5x	40
	Scp6x	16
	Scpax	40
	Scpbx	16
	Scpcx	40
	Scpdx	16
	Scpnrex	8
	Scpnrfx	4
	Scpnrgx	40
	Scpnrhx	16
0.6	Scp4x	30
	Scp5x	30
	Scp6x	12
	Scpax	30
	Scpbx	12
	Scpcx	30
	Scpdx	12
	Scpnrex	6
	Scpnrfx	3
	Scpnrgx	30
	Scpnrhx	12
0.4	Scp4x	20
	Scp5x	20
	Scp6x	8
	Scpax	20
	Scpbx	8
	Scpcx	20
	Scpdx	8
	Scpnrex	4
	Scpnrfx	2
	Scpnrgx	20
	Scpnrhx	8

**Table 3:** Results with binary encoding.

Tightness Ratio	Instance Set	One-Point Crossover Conservative Repair		Uniform Crossover Conservative Repair		*p*-Value	One-Point Crossover Random Repair		Uniform Crossover Random Repair		*p*-Value
		Average	Time *	Average	Time *		Average	Time *	Average	Time *	
0.8	Scp4x	183.41	3.6	195.19	5.4	2.80 × 10^−21^	188.72	5.7	196.16	5.0	1.04 × 10^−16^
	Scp5x	185.62	5.0	197.90	10.6	6.45 × 10^−18^	191.49	8.0	198.81	9.8	1.38 × 10^−16^
	Scp6x	170.73	3.1	177.27	5.1	9.78× 10^−15^	168.83	4.4	179.29	4.3	1.13 × 10^−21^
	Scpax	268.55	7.1	287.35	18.1	6.12 × 10^−25^	278.11	14.7	289.72	16.9	1.20 × 10^−19^
	Scpbx	247.73	6.8	258.12	19.5	6.86 × 10^−25^	249.27	13.8	262.26	14.8	1.24 × 10^−17^
	Scpcx	344.37	9.6	369.04	28.4	7.78 × 10^−28^	357.41	19.2	372.36	25.2	1.80 × 10^−17^
	Scpdx	318.18	8.9	329.05	30.2	4.22 × 10^-13^	318.06	19.5	334.39	24.0	3.59 × 10^−16^
	Scpnrex	366.33	10.5	373.31	45.1	1.18 × 10^−7^	363.55	23.4	378.43	38.5	6.68 × 10^−15^
	Scpnrfx	356.83	10.3	360.22	46.3	2.93 × 10^−3^	354.57	24.6	363.09	35.7	3.40 × 10^−10^
	Scpnrgx	762.38	23.1	810.48	128.0	2.77 × 10^−26^	791.27	66.3	819.97	141.9	3.29 × 10^−20^
	Scpnrhx	713.31	20.8	732.47	144.6	4.48 × 10^−16^	716.28	77.1	743.23	168.2	6.27 × 10^−21^
0.6	Scp4x	163.96	3.8	174.44	4.5	5.03 × 10^−17^	169.79	5.6	176.31	7.1	1.04 × 10^−12^
	Scp5x	166.66	4.6	179.24	9.2	3.51 × 10^−25^	173.48	8.1	181.87	14.4	5.52 × 10^−14^
	Scp6x	149.11	3.0	153.73	4.6	5.27 × 10^−10^	152.00	4.3	155.48	6.2	1.26 × 10^−4^
	Scpax	240.75	6.9	256.74	23.3	8.49 × 10^−22^	250.27	13.5	261.54	23.3	1.20 × 10^−13^
	Scpbx	215.96	6.6	223.06	24.4	5.16 × 10^−10^	220.55	13.6	227.51	20.6	2.01 × 10^−9^
	Scpcx	304.84	9.2	325.85	37.1	6.25 × 10^−24^	317.47	17.9	331.65	35.0	1.75 × 10^−15^
	Scpdx	274.95	8.3	282.91	37.4	5.15 × 10^−10^	280.47	16.8	287.85	28.8	3.68 × 10^−9^
	Scpnrex	310.39	10.5	315.32	55.7	4.41 × 10^−5^	315.23	23.1	319.58	38.0	2.40 × 10^−5^
	Scpnrfx	300.01	10.5	302.39	58.0	3.87 × 10^−2^	303.78	18.5	305.95	36.0	2.10 × 10^−2^
	Scpnrgx	657.22	23.0	694.44	164.2	1.75 × 10^−25^	683.50	74.7	709.42	130.9	7.79 × 10^−17^
	Scpnrhx	604.66	20.6	617.16	186.4	3.78 × 10^−8^	613.69	76.2	626.77	101.3	2.19 × 10^−8^
0.4	Scp4x	131.67	2.9	140.35	2.2	2.12 × 10^−18^	136.56	2.8	140.33	6.3	4.47 × 10^−10^
	Scp5x	135.07	4.2	146.09	2.6	2.96 × 10^−23^	140.62	4.3	145.99	12.5	1.79 × 10^−10^
	Scp6x	116.19	2.6	120.16	2.1	6.29 × 10^−11^	118.19	2.2	119.89	5.3	4.33 × 10^−4^
	Scpax	194.18	7.3	209.70	26.0	1.04 × 10^−21^	201.23	7.4	209.34	17.4	1.19 × 10^−11^
	Scpbx	167.82	7.1	176.21	27.1	2.11 × 10^−12^	172.24	6.6	176.22	17.8	8.73 × 10^−6^
	Scpcx	243.81	8.7	262.97	14.7	1.15 × 10^−23^	253.37	11.8	263.24	32.7	6.31 × 10^−11^
	Scpdx	211.58	7.9	220.80	26.0	2.82 × 10^−15^	216.45	13.5	220.73	28.3	6.19 × 10^−4^
	Scpnrex	233.95	10.3	240.55	38.8	2.64 × 10^−10^	238.29	21.8	240.55	31.9	9.82 × 10^-3^
	Scpnrfx	224.72	10.3	229.89	40.0	6.42 × 10^−5^	228.43	13.2	229.59	22.3	2.63 × 10^−1^
	Scpnrgx	510.30	20.5	548.71	113.8	3.49 × 10^−25^	531.73	69.9	548.64	150.5	3.66 × 10^−12^
	Scpnrhx	455.80	18.7	471.26	117.1	6.92 × 10^−12^	463.71	83.4	471.14	133.6	3.35 × 10^−6^

The bold values represent the best objective value or the shortest running time among the compared methods. * CPU Time in seconds.

**Table 4:** Results with integer encoding.

Tightness Ratio	Instance Set	One-Point Crossover Conservative Repair		Uniform Crossover Conservative Repair		*p*-Value	One-Point Crossover Random Repair		Uniform Crossover Random Repair		*p*-Value
		Average	Time *	Average	Time *		Average	Time *	Average	Time *	
0.8	Scp4x	180.39	3.5	191.46	2.5	4.01 × 10^−15^	185.95	3.4	177.61	4.8	8.33 × 10^−14^
	Scp5x	181.86	2.8	193.21	2.5	5.70 × 10^−7^	188.00	3.1	181.43	4.4	1.72 × 10^−9^
	Scp6x	169.87	2.5	176.66	1.5	4.62 × 10^−14^	144.62	3.0	176.01	3.2	6.13 × 10^−38^
	Scpax	263.40	4.6	283.23	3.5	7.53 × 10^−20^	272.05	4.9	266.43	6.4	2.47 × 10^−5^
	Scpbx	246.57	4.0	257.15	2.4	4.02 × 10^−14^	225.33	4.1	256.15	4.6	4.51 × 10^−39^
	Scpcx	337.83	5.9	364.61	4.3	8.24 × 10^−25^	349.81	5.3	342.84	8.6	1.48 × 10^−5^
	Scpdx	315.93	5.1	328.41	2.8	2.11 × 10^−12^	317.03	5.1	327.29	5.8	4.71 × 10^−12^
	Scpnrex	366.17	5.7	372.01	2.8	5.04 × 10^−5^	367.07	5.6	371.65	5.7	2.65 × 10^−3^
	Scpnrfx	357.79	4.7	358.93	2.6	2.89 × 10^−1^	357.52	4.8	358.69	4.8	2.91 × 10^−1^
	Scpnrgx	748.79	11.7	807.32	8.8	8.20 × 10^−30^	773.33	11.9	773.01	16.4	3.87 × 10^−1^
	Scpnrhx	707.91	10.4	729.73	8.9	2.40 × 10^−17^	712.26	11.7	729.47	8.2	2.95 × 10^−10^
0.6	Scp4x	161.46	1.6	174.29	3.9	1.16 × 10^−19^	165.93	2.9	168.17	3.6	7.63 × 10^−2^
	Scp5x	163.77	1.5	179.00	3.6	5.07 × 10^−21^	168.61	2.4	172.80	3.6	1.47 × 10^−4^
	Scp6x	148.59	1.2	153.13	2.3	4.17 × 10^−9^	149.53	2.2	152.31	2.2	3.20 × 10^−4^
	Scpax	236.03	2.0	256.75	4.8	3.00 × 10^−23^	242.60	3.6	251.19	4.8	7.71 × 10^−7^
	Scpbx	214.72	1.8	222.79	3.4	2.62 × 10^−11^	215.85	3.2	221.51	3.2	2.08 × 10^−6^
	Scpcx	298.75	2.4	325.55	5.4	1.52 × 10^−23^	307.89	4.6	319.44	6.4	2.65 × 10^−10^
	Scpdx	272.88	22.6	281.37	4.5	4.07 × 10^−12^	274.35	4.5	282.01	4.2	8.37 × 10^−8^
	Scpnrex	311.15	2.4	314.79	4.2	1.22 × 10^−3^	311.85	4.3	314.32	4.8	4.35 × 10^−2^
	Scpnrfx	301.25	2.2	301.95	4.6	5.35 × 10^−1^	301.53	3.7	301.97	5.3	5.92 × 10^−1^
	Scpnrgx	647.72	5.4	694.89	11.1	1.82 × 10^−26^	665.05	10.0	691.83	12.7	1.18 × 10^−11^
	Scpnrhx	600.34	4.8	615.69	6.5	1.59 × 10^−12^	602.57	9.6	614.51	8.4	1.97 × 10^−6^
0.4	Scp4x	130.64	1.3	138.29	2.5	3.06 × 10^−15^	132.76	2.3	137.08	2.5	5.70 × 10^−7^
	Scp5x	133.39	1.0	143.11	2.4	3.03 × 10^−18^	135.65	2.0	141.65	2.2	5.74 × 10^−9^
	Scp6x	116.33	0.9	118.42	1.7	2.83 × 10^−3^	116.54	1.9	118.39	1.4	3.41 × 10^−3^
	Scpax	191.93	1.3	204.32	3.9	3.51 × 10^−16^	194.89	2.7	203.47	2.7	1.60 × 10^−11^
	Scpbx	167.96	1.2	171.34	2.7	2.13 × 10^−4^	168.73	2.2	171.36	2.1	2.96 × 10^−3^
	Scpcx	240.32	1.8	255.59	4.1	7.55 × 10^−16^	244.89	3.7	254.33	3.6	3.61 × 10^−8^
	Scpdx	210.52	1.8	215.30	3.2	1.16 × 10^−3^	212.19	3.2	215.09	2.9	1.05 × 10^−2^
	Scpnrex	234.47	1.7	236.19	3.2	9.25 × 10^−2^	235.55	3.4	236.29	3.3	4.45 × 10^−1^
	Scpnrfx	225.87	1.6	226.07	3.2	5.19 × 10^−1^	226.09	2.8	225.43	3.7	4.92 × 10^−1^
	Scpnrgx	505.53	4.0	531.63	8.4	2.53 × 10^−16^	512.51	8.2	530.95	8.0	1.37 × 10^−11^
	Scpnrhx	455.25	3.7	462.76	7.5	6.31 × 10^−5^	456.15	6.0	462.25	6.8	2.99 × 10^−3^

The bold values represent the best objective value or the shortest running time among the compared methods. * CPU Time in seconds.

## Data Availability

The data presented in this study are available in [OR-Library] at [https://people.brunel.ac.uk/~mastjjb/jeb/orlib/scpinfo.html] (accessed on 15 October 2022), reference number [33].

## Appendix A. Experimental Analysis of Crossover Operators in Binary-Encoding with Random Repair for scp4x

We conducted an additional experiment on the SCP4 instance with a tightness ratio of 0.8, using binary encoding and random repair. This experiment aimed to investigate the performance of various crossover ensembles. To observe the effect of combining different crossover methods, we employed one-point crossover (OX1), two-point crossover (OX2), and uniform crossover (Uniform). The seeds used for these ensemble crossovers were different from those in the main experiments.

Table A1 presents the average and standard deviation of performance and computation time for SCP4x, evaluated using various crossover combinations. In the crossover ensembles, a random number between 0 and 1 was generated each time an offspring was created, and a crossover operator was selected probabilistically based on this value. As shown in Table A1, the OX2 + Uniform ensemble achieved the best performance, while using only OX1 resulted in the worst performance. Notably, while OX1 and OX2 fail to ensure population diversity in binary encoding, leading to poorer performance compared to Uniform, their performance significantly improves when used in conjunction with Uniform. These results suggest that an appropriate combination of crossover operators can significantly enhance the performance of a genetic algorithm compared to using a single crossover operator.

**Table A1:** Performance of different crossover ensembles using binary encoding and random repair on SCP4x with a tightness ratio of 0.8.

Methods	Average	std	Time *	std
OX1 + OX2 + Uniform	193.85	1.67	35.20	19.68
OX1 + OX2	190.31	2.60	25.87	1.44
OX1 + Uniform	194.04	2.09	41.40	2.31
OX2 + Uniform	194.70	1.87	40.35	2.25
OX1	188.98	2.77	24.43	1.37
OX2	191.24	2.25	26.87	1.57
Uniform	192.97	2.96	40.27	2.73

* CPU Time in seconds.
